# Consciousness: A Neurological Perspective

**DOI:** 10.3233/BEN-2011-0322

**Published:** 2011-03-29

**Authors:** Andrea E. Cavanna, Sachin Shah, Clare M. Eddy, Adrian Williams, Hugh Rickards

**Affiliations:** ^1^Department of NeuropsychiatryUniversity of Birmingham and BSMHFTBirminghamUK; ^2^Department of NeuropsychiatryInstitute of NeurologyUCLLondonUK; ^3^University of Birmingham Medical SchoolBirminghamUK; ^4^Division of NeurosciencesUniversity of Birmingham and UHBBirminghamUK

**Keywords:** Arousal, awareness, coma, consciousness, epilepsy, neurology, sleep

## Abstract

Consciousness is a state so essentially entwined with human experience, yet so difficult to conceptually define and measure. In this article, we explore how a bidimensional model of consciousness involving both level of arousal and subjective awareness of the contents of consciousness can be used to differentiate a range of healthy and altered conscious states. These include the different sleep stages of healthy individuals and the altered states of consciousness associated with neurological conditions such as epilepsy, vegetative state and coma. In particular, we discuss how arousal and awareness are positively correlated in normal physiological states with the exception of REM sleep, while a disturbance in this relationship is characteristic of vegetative state, minimally conscious state, complex partial seizures and sleepwalking.

## 1. Two components of consciousness: arousal and awareness

For something experienced so intimately by humans, consciousness has nonetheless proven a difficult concept to explain [26]. Consciousness is an ambiguous term, with multiple definitions. Zeman et al. identified several different senses in which the term is used [80], two of which have been of particular interest in the investigation of consciousness: consciousness as arousal and consciousness as awareness [79].

### *1.1. Arousal*

In clinical practice consciousness is typically equated with arousal (i.e. wakefulness, alertness, or vigilance), referring to the ability of an individual to respond to stimuli in the integrated manner expected in the waking state [79]. In healthy individuals, varying degrees of arousal correspond to a range of conscious states that extend from deep dreamless sleep, through drowsiness to alert wakefulness [64]. Consciousness in this sense can be evaluated objectively using behavioural criteria such as those specified in the Glasgow Coma Scale (GCS) [74].

Electroencephalogram (EEG) recordings from the cerebral cortex reveal different wave patterns of electrical activity in the brain corresponding to different levels of arousal [8]. Activity is typically divided into four basic types, according to frequency of waveforms per second (hertz). Beta activity is low amplitude fast activity occurring at frequencies of 13–30 Hz, characterising an "active EEG" associated with mental exertion. Alpha activity, at frequencies of 8–13 Hz, characterises the "passive EEG", and is the predominant background activity during the relaxed waking state when the individual's eyes are closed. Theta activity is a slower rhythm of 4–7 Hz, present in states of drowsiness. Even slower frequencies of less than 4 Hz are seen in delta activity, associated with sleep. Thus it is evident that increasing levels of arousal are normally associated with increasing frequency of electrical activity in the cerebral cortex. This is caused by a reduction in the extent and degree of cortical synchronisation.

Arousal is a function of the ascending reticular activating system (ARAS), a functional component of the

ISSN 0953-4180/11/$27.50 © 2011 – IOS Press and the authors. All rights reserved complex neuronal network within the reticular formation of the upper brainstem. The ARAS contains two major axes: the reticulothalamocortical pathway and extrathalamic pathways. Activation of the reticulothalamocortical pathway promotes cortical arousal by facilitating the transthalamic passage of sensory information towards the cerebral cortex [59]. The cholinergic system of the ARAS provides the main input to the thalamic nuclei from the upper brainstem [65]. The intralaminar and reticular nuclei are the thalamic components most associated with this pathway of the ARAS [8]. Meanwhile extrathalamic pathways activate the cortex via a series of direct inputs originating in the brainstem and basal forebrain and collectively exert a large influence on arousal [59].

## *1.2. Awareness*

Consciousness as awareness refers to the content of experience: awareness of the environment and the self. An individual who is conscious will be conscious of something, and it is in this sense that they have awareness. The content of an individual's subjective experience is comprised of their sensations, thoughts, emotions, memory, imagination and other major psychological processes [79].

The contents of consciousness are associated with activity in specific cortical areas. For example, awareness of visual movement is associated with activity in visual area V5, while awareness of colour is associated with activity in visual area V4 [76]. Focal cortical lesions in these areas have led to akinetopsia and achromatopsia, respectively, in affected individuals [77,78].

How the level of arousal and the contents of consciousness interact is a complex matter and yet to be fully explained. It has been demonstrated that the contents of consciousness can vary independently of the level of consciousness [64]. However, in most conditions the level of consciousness can greatly influence the contents of consciousness. As might be expected, an increase in the level of arousal leads to an increase in the extent and quality of conscious experience. In normal physiological states, arousal and content are positively correlated (Fig. 1). An exception is the dream-state seen in "rapid eye movement" (REM) sleep, where a low level of arousal is associated with vivid sensory imagery [30]. The positive correlation between arousal and content is also disrupted in certain pathological conditions. High levels of arousal, for example, may be associated with poor content of experience, as seen in limbic status epilepticus [60].

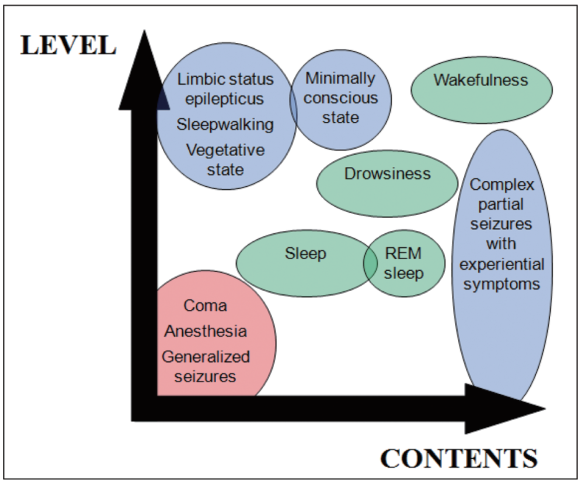

Fig. 1. Simplified representation of consciousness as a bidimensional model, along the two axes of Level (Y) and Contents (X). Normal physiological states (green) show positive correlation between level and content of consciousness, with the exception of the dream state in REM sleep. Abnormal states (red) such as coma, anaesthesia and generalised seizures represent a loss of consciousness where both arousal and awareness is low or absent. Dissociated states of consciousness (blue) include vegetative state, sleepwalking and limbic status epilepticus, in which the individual exhibits a high level of arousal but a low awareness of themselves and their surroundings. Minimally conscious state (MCS) is differentiated from vegetative state as individuals in MCS can demonstrate limited levels of awareness. Complex partial seizures with experiential symptoms involve a variable level of arousal, while the contents of consciousness are highly vivid, characterized by seizure-induced experiential phenomena.

In a healthy individual, levels of arousal will vary during wakefulness depending on the individual's activity. Variations in arousal during the day correlate with levels of activity in the structures of the midbrain and thalamus that regulate conscious states [79]. A positron emission tomographic (PET) study demonstrated activation of the midbrain reticular formation and thalamic intralaminar nuclei when individuals transitioned from a relaxed awake state to an attention-demanding reaction-time task, confirming the role of these areas of the brain and brainstem in arousal and vigilance [40]. Meanwhile, a decrease in midbrain and thalamic activation was observed during a tedious auditory vigilance task, associated with increasingly slow reaction time and increasingly slow wave activity recorded on EEG [63]. The authors suggested that the observed time-related changes in reaction time and EEG activity are both related to changes in the level of arousal (alertness) and that regional cerebral blood flow (rCBF) changes in the thalamus-related neural circuitry represent a brain correlate of such changes.

The lowest level of arousal in a healthy individual occurs during sleep, a state of reversible unconsciousness. While it is inherently difficult to prove complete absence of consciousness in state studies, there is no question that deep sleep is much less conscious than full, responsive wakefulness [5]. There is a distinction between the two main phases of sleep: “slow wave sleep”, which is associated with a high proportion of delta activity in the EEG, and REM sleep, during which the EEG resembles that seen during wakefulness. Paradoxically, individuals are more difficult to arouse during REM sleep [79]. Despite the low level of arousal, REM sleep is associated with the dreaming state and a raised content of consciousness, the exception to the otherwise positive correlation between level and content of consciousness in normal physiological states [51] (Fig. 1).

Global cerebral glucose metabolism falls around 20% in slow wave sleep, and rises back up during REM sleep (sometimes surpassing even waking levels) [20]. During slow wave sleep, there are marked decreases of rCBF in the pons, mesencephalon, thalamic nuclei and basal forebrain, reflecting the close implication of these structures in the generation of slow wave sleep rhythms [54]. It appears that reduced rCBF in these subcortical structures facilitates synchronization in their diffuse cortical projections, resulting in the slow signal observed on the scalp EEG. On the other hand, in REM sleep rCBF increases in the rostral brainstem, thalamus and limbic regions, while there are decreases in rCBF in prefrontal and posterior cingulate cortex, and in some regions of parietal cortex [55].

## 2. Abnormal states of reduced arousal and awareness

### 2.1. Coma

Coma is a state of unconsciousness characterised by an absence of arousal due to a failure of the ARAS [73]. In this condition the patient cannot be aroused, and has no awareness of themselves or their surroundings. Coma can be objectively assessed using the GCS, which arbitrarily defines coma as a failure to open eyes in response to verbal command, perform no better than weak flexion, and utter only unrecognisable sounds in response to pain [7]. Structural brain lesions that produce coma must include ARAS structures or render the cerebral cortex diffusely dysfunctional; thus coma can result from diffuse bihemispheric cortical or white matter injury, or from focal brainstem lesions that affect the pontomesencephalic tegmentum or paramedian thalami bilaterally [50].

The metabolic rate of glucose in the thalamus, brain stem, and cerebellum can be significantly lower in comatose in comparison to noncomatose patients, after traumatic brain injury (TBI) [36]. On average, grey-matter metabolism is 50–70% that of the normal range in comatose patients of traumatic or hypoxic origin. However, it has been shown that level of consciousness (as measured with the GCS) can be poorly correlated with cerebral glucose metabolism within the first month following TBI [9].

### 2.2. Anaesthesia

Anaesthesia is a state of pharmacologically induced unconsciousness. There is evidence that the loss of consciousness induced by some anaesthetics is associated with selective depression of thalamic function, linking the mechanisms of anaesthesia and sleep. Administration of propofol to subjects, for example, preferentially decreases rCBF in brain regions implicated in the regulation of arousal, such as the thalamus and midbrain [28]. Patients anaesthetised to the point of unresponsiveness show a reduction in brain metabolism similar to that of comatose patients, with values reaching 28% of the normal range under propofol anaesthesia [3].

These altered states of consciousness continue the positive correlation between arousal and content: both dimensions of consciousness are impoverished in coma and under anaesthesia (Fig. 1).

## 3. States of dissociated arousal and awareness

There are certain pathological states in which the association between arousal and awareness is lost (Fig. 1). In conditions where arousal is present in the absence of awareness, the individual will appear to be in a zombie-like state, seemingly awake but with no behavioural evidence of “voluntary” or “willed” behaviour [51]. In contrast, conditions such as complex partial seizures may experience vivid content of consciousness while varying in level of arousal. This section shall briefly cover the vegetative state, minimally conscious state, epileptic seizures and sleepwalking.

### *3.1. Vegetative state*

Patients in a comatose state may progress to a vegetative state, a state of "wakeful unawareness". This transition is typically marked by when a comatose patient regains their sleep/wake cycle. The vegetative state is often described clinically as loss of function of the cortex while the function of the brainstem and allied structures (encompassing the pedunculopontine reticular formation, the hypothalamus, and the basal forebrain) is preserved [1]. The functional preservation of these structures maintains arousal and autonomic functions in these patients [54].

Jennet and Plum chose the term "vegetative state" as a description of the patient's behaviour, referring to the definition of "vegetative" as describing "an organic body capable of growth and development but devoid of sensation and thought" [37]. A vegetative state persisting for a month following TBI or non-traumatic brain damage is referred to as a "persistent vegetative state," while a vegetative state that persists for 3 months following a non-traumatic brain injury or 12 months following TBI is regarded as irreversible and thus termed a "permanent vegetative state" [71]. Given sufficient medical care, including artificial hydration and nutrition, patients in a vegetative state may survive for many years [51].

Patients in the vegetative state may be aroused by painful or salient stimuli, but they demonstrate no clear signs of conscious perception or deliberate action [79]. Stereotyped responses to external stimuli, such as grimacing, are thought to result from preserved brainstem circuits and limbic cortical regions [54]. It may be difficult for patients' family members to accept that such reflexive movements do not mean the patient is conscious: this highlights the misconception that arousal implies awareness. Patients in a vegetative state show no signs of being aware of themselves or their environment [62].

Studies of patients in a vegetative state have shown that the relationship between global levels of brain function and the presence of awareness is not absolute. In the vegetative state, PET studies have demonstrated global brain metabolic activity decreases to around 50% of normal levels [48], a similar decrease to that seen in anaesthetised subjects and in deep sleep. However, in certain cases where patients recovered from vegetative state and regained consciousness, global glucose utilisation levels remained essentially the same after recovery as they were during the vegetative state, indicating that the recovery of consciousness was related to a modification of the regional distribution of brain function rather than to the global resumption of cerebral metabolism [49]. Furthermore, there have been reports of fully awake healthy individuals with levels of global brain metabolism similar to those seen in some patients in a vegetative state [51], and patients in a vegetative state who exhibit close to normal global cortical metabolism [68]. Thus it appears specific areas of the brain are important for awareness. Such regions may be identified through functional neuroimaging of patients in vegetative state and comparison with healthy conscious controls.

Studies searching for regions of metabolic dysfunction in the brains of patients in a vegetative state as compared with the brains of resting conscious healthy controls have identified metabolic dysfunction in a "wide frontoparietal network", encompassing the polymodal associative cortices: lateral and medial frontal regions bilaterally, parieto-temporal and posterior parietal areas bilaterally, posterior cingulate and precuneal cortices [51]. Not only is activity in the frontoparietal network seemingly related to awareness, but so is functional connectivity within the network, and with the thalami. Compared to healthy controls, patients in a vegetative state appear to have functional disconnections in long-range cortico – cortical (between laterofrontal and midline-posterior areas) and cortico – thalamo – cortical (between non-specific thalamic nuclei and lateral and medial frontal cortices) pathways [46, 48]. Recovery from the vegetative state is associated with a functional restoration of such pathways [51].

Functional neuroimaging reveals the brain areas that are activated in patients in a vegetative state when external stimulation is applied to them. A pain stimulus from electrical stimulation resulted in activation in the midbrain, contralateral thalamus, and primary somatosensory cortex in all patients from a sample of 15 patients in a vegetative state [47]. However, in contrast to control subjects, higher-order areas of the pain matrix (secondary somatosensory, insular, posterior parietal and anterior cingulate cortices) were not activated. In addition, the activated primary somatosensory cortex was isolated from the frontoparietal network, which is thought to be required for conscious perception [51]. Similar results were seen with auditory stimulation in patients in a vegetative state: the stimulation activated primary auditory cortices, but did not activate higher-order multimodal areas from which they were disconnected [18,45]. In general, stimuli will activate primary cortical areas but do not activate secondary cortical areas (believed to be necessary for awareness), which are functionally disconnected from the primary cortical regions [10]. In functional neuroimaging, the absence of activation of higher order multimodal association cortices that provide the brain's integrated, distributed, neuronal networks invariably correlates with lack of awareness of patients in a vegetative state [36].

### 3.2. *Minimally conscious state*

The minimally conscious state (MCS) is sometimes distinguished from the vegetative state. Patients may progress into a MCS following a coma. Patients in a MCS appear similar to vegetative state patients, exhibiting an inability to communicate consistently, but in contrast to vegetative state patients, they show limited yet clear evidence of awareness, on a reproducible or sustained basis, by demonstrating at least one of the following: following simple commands, gestural or verbal yes/no response, intelligible speech, or purposeful behaviour (including reactions to environmental stimuli that are not reflex actions). Recovery from the MCS is defined as the ability to communicate or use objects functionally [31]. MCS is, like the vegetative state, a state of disconnected arousal and content: like in the vegetative state, the patient is fully aroused, but unlike in the vegetative state, the patient is able to demonstrate a limited level of awareness, though not at a level that would be expected from a healthy individual at the same level of arousal.

Overall cerebral metabolism in MCS is comparable to that of vegetative patients [54]. The precuneus and posterior cingulate cortex of patients in MCS show an intermediate level of metabolism, lower than in healthy conscious individuals, but higher than in patients in a vegetative state [44]. Thus it is hypothesised that these regions represent part of the neural network subserving consciousness [54]. In MCS patients, rather than merely activating primary auditory corticies as in the vegetative state, auditory stimulation also activated auditory associative areas, suggesting the occurrence of more complex processing [18]. In addition, compared to patients in a vegetative state, patients in a MCS demonstrated a stronger functional connectivity between the secondary auditory cortex and temporal and prefrontal association cortices [18]. This again demonstrates the importance of activity in association cortices for awareness.

### 3.3. *Epileptic seizures*

Epileptic seizures are also associated with a state of impaired consciousness in which arousal and awareness appear dissociated [21,24,25]. Impairment of consciousness is the defining feature that distinguishes complex partial seizures from simple partial seizures [60]. In addition to complex partial seizures, two other types of seizures are known to cause impairment of consciousness: generalized tonic–clonic (GTC) seizures and absence seizures [15]. Such a loss of consciousness is judged through the patient's responsiveness during the ictal state. While responsiveness is reduced in both complex partial seizures and generalized seizures, the different seizure types have different effects on the individual's consciousness, due to their different effects on the brain. Generalized seizures (including GTC seizures and absence seizures) are characterized by abnormal electrical activity in both hemispheres and complete loss of consciousness, while complex partial seizures typically cause disturbances limited to sensory processes, perception, memory, or attention, resulting in motor or sensory aphasia, or transient inattention, which may be mistaken for loss of consciousness [75]. Though the three types of seizure act differently, the same anatomical networks have been implicated in impaired consciousness, through either abnormal increases or abnormal decreases in neuronal activity [22,74].

Generalised seizures can be either primarily generalised, which have no inciting focus but begin bilaterally in both hemispheres of the cerebral cortex, or secondarily generalised, with onset in one focus of the brain which then spreads to other regions [10]. Both primarily and secondarily generalized seizures are invariably associated with a complete and transient loss of consciousness. GTC seizures and absence seizures are thus the most common causes of epilepsy-induced loss of consciousness [18].

Absence seizures are characterised by an interruption of the individual's behaviour, with staring and unresponsiveness, along with possible spasms. Typical absence seizures are characterised on an EEG by large-amplitude spike – waves at a frequency of approximately 3 Hz. This is thought to result from abnormal network oscillations involving the cortex of the two hemispheres and the thalamic nuclei, which represent the target of the brainstem reticular activating projections [23]. Individuals in this state lack subjective experience, as the seizure entails a blank episode with no awareness or content of consciousness [23].

Functional magnetic resonance imaging (fMRI) with simultaneous EEG recordings have confirmed that generalized spike – wave seizures selectively involve certain networks while sparing others [2,34,42,43,67].

These studies have shown activity is decreased in the medial prefrontal cortex, anterior cingulate cortex, and precuneus – posterior cingulate cortex, and increased in the upper brainstem and midline, mediodorsal and intralaminar thalamic nuclei. The lateral frontoparietal association network shows both increased and decreased activity ictally and decreased activity postictally. It has been hypothesised [15,16,44] that loss of consciousness in absence seizures is caused by a disruption of the normal information processing in specific brain regions (bilateral association cortex and related subcortical structures) rather than resulting from the involvement of the entire brain.

GTC seizures are widespread neuronal discharges that include abnormal activity throughout large areas of the brain [18]. A "tonic" phase lasts 10-20 seconds during which there is high-frequency EEG activity, along with sustained muscle contractions seen in the individual. The "clonic" phase then follows in which the individual's limbs contract rhythmically as brain activity settles into poly-spike-and-wave EEG discharges, which correspond with the activity of large assemblies of neurons, which alternately fire in strong bursts and turn silent [23]. Seizure activity as recorded on the EEG usually ends after around two minutes, after which the individual is typically lethargic and unresponsive for several minutes [18]. As such a large brain area is involved in generalized seizures, nearly all GTC seizures result in impaired consciousness [14].

Recent single photon emission computed tomography (SPECT) imaging studies of cerebral blood flow in secondary GTC seizures have shown that such seizures do not affect the entire brain homogeneously but may instead present with focal involvement, often in regions of seizure onset [14,17,52,69]. During secondary GTC seizures, activity decreased in the medial prefrontal cortex, anterior cingulate cortex, and precuneus–posterior cingulate cortex, and increased in the upper brainstem and the midline, mediodorsal and intralaminar thalamic nuclei [16,22,43,58]. The lateral frontoparietal association network showed increased activity ictally and decreased activity postictally (when the patient remained unconscious). The regions most intensely involved in cerebral blood flow increases during GTS seizures were the bilateral frontal and parietal association areas, whereas the intervening primary sensory and motor cortices were relatively spared. Similar results have been shown in investigations of primary GTS seizures [35].

While the generalized seizures mentioned above are characterized by complete unresponsiveness and absence of any ictal subjective experience, during complex partial seizures the level and the contents of ictal consciousness can each be affected to varying degrees.

Partial seizures originate in specific areas of the cortex and then either remain localised there or spread to other areas of the brain. They are classified further as simple partial seizures, with retained consciousness, or complex partial seizures, defined by loss of consciousness [10]. Temporal lobe seizures are a common type of partial seizure and can likewise be either simple or complex. Complex partial temporal lobe seizures typically begin with focal premonitory phenomena such as fear, rising abdominal sensation or lip-smacking automatisms ('epileptic aura') [4]. Though consciousness may initially be spared, the individual progressively loses contact with the environment, exhibits a fixed stare, and can become unresponsive. The individual will commonly demonstrate stereotyped, automatic behaviours. Complex partial seizures usually last from 15 seconds to 3 minutes. In patients with temporal lobe epilepsy, during the seizure, localized abnormal rhythmic activity usually arises in the temporal lobe, seen as 5–7 Hz rhythmic activity on scalp EEG recordings [72]. With complex partial seizures, postictal behaviour often continues to show impairment of consciousness for several minutes, complete with confusion and often amnesia of the event [10].

Complex partial seizures of temporal lobe origin are typically associated with intense subjective experiences, termed "experiential phenomena", which typically encompass mnemonic, affective or perceptual phenomena, such as complex illusions and hallucinations, which commonly relate to the patient's past experience [32]. Affective phenomena include feelings of fear, guilt, sadness, euphoria, joy or excitement, along with symptoms of depersonalisation and derealisation [67]. It has been proposed that such affective phenomena are the result of activation of the amygdala and other limbic structures [29]. Thus, focal seizures are thought to modulate the contents of ictal conscious state in medial temporal lobe epilepsy [45].

A recent SPECT imaging study [11] found complex partial temporal lobe seizures produced CBF increases in the temporal lobe (the seizure focus), followed by increases in bilateral midline subcortical structures, including the mediodorsal thalamus and upper brainstem. These changes were accompanied by simultaneous marked decreases in CBF in the frontal and parietal association cortices (lateral prefrontal, anterior cingulate, orbital frontal, and lateral parietal cortex). Simple partial seizures, which involve no loss of consciousness, were associated with CBF changes mainly restricted to the temporal lobe, and were not accompanied by the widespread impaired function of the frontoparietal association cortices as seen in complex partial seizures. Pronounced slowing in bilateral frontal and parietal association cortices in patients undergoing complex partial temporal lobe seizures is further evidenced by intracranial EEG recordings taken of these patients [12]. Such findings support the hypothesis that loss of consciousness during temporal lobe seizures may result from focal seizures arising in the medial temporal lobe spreading to midline subcortical structures (upper brainstem – diencephalic activating systems), disrupting their activating function, and thus secondarily leading to widespread inhibition of the frontoparietal cortex [53,61].

Complex partial seizures demonstrate a state in which content and level of consciousness are dissociated: the level of arousal is hugely variable, yet the contents of consciousness are highly vivid, characterized by seizure-induced experiential phenomena.

Meanwhile, limbic status epilepticus presents the opposite situation: there is a lack of any subjective experience, accompanied by a degree of awareness of the external environment. Limbic status epilepticus, formally known as psychomotor status, is a state of prolonged complex partial seizure. Patients in this state have been described as zombie-like, performing activities in an automatic fashion while completely unaware of their purposeful actions. Such "zombie mode" [41] activity is present in healthy individuals, for example the automatic nature of driving a car while the driver's attention is diverted elsewhere.

Such behaviour may be a result of "forced attention": the narrowing of the focus of attention and the absence of voluntary control over the direction of attention, a constant feature of complex partial seizures [38]. It has been suggested that this phenomenon is due the spreading of pathological electrophysiological discharges to the frontal networks involved in attentional control [33, 75]. Thus individuals undergoing limbic status epilepticus may be somewhat aware of their environment, but are focusing their attention on the vivid experiential phenomena induced by the electrical stimulation of the temporal lobe [31].

### 3.4. *Sleepwalking*

A further case of dissociation is seen in individuals who experience sleepwalking (somnambulism). Much like patients in limbic status epilepticus, sleepwalkers will carry out actions in a "zombie-like" automatic fashion while showing no awareness of their purposeful actions. Sleepwalking individuals will perform various complex motor behaviours, including walking, during slow wave sleep [19]. Patients typically stand up and walk around quietly and aimlessly. In a single patient studied using SPECT, it was observed that large areas of frontal and parietal association cortices remained deactivated during sleepwalking in comparison to healthy subjects during wakefulness [6]. Similar reductions in CBF are seen in this frontoparietal network in patients suffering from complex partial seizures, absence seizures, and in vegetative state [51].

## 4. Conclusions

A bidimensional model measuring consciousness both as arousal and awareness is critical when working with neurological conditions affecting the conscious state. While arousal and content of consciousness are typically correlated in healthy individuals, they are dissociated in certain conditions. Studying these conditions will help shed light on the neural substrates of both arousal and awareness. By comparing brain activity when consciousness is normal and impaired, the systems implicated in the control of consciousness can effectively be modelled. For example, it is evident that frontoparietal areas are necessary for conscious perception: pain signals do not spread beyond the sensory cortex to the frontoparietal areas in vegetative state patients, and are thus not consciously perceived, despite the patient being fully aroused. Regional metabolism is high in the frontoparietal cortex in the conscious resting state [57,66], while low in deep sleep [56], under general anaesthesia [39] and during vegetative state [49] and epileptic loss of consciousness [44].

Further neuroimaging research involving patients in a vegetative state could yield crucial information about how consciousness is mapped in the brain, as well as helping to quantify the extent of awareness of these patients. This knowledge can then be applied to help detect residual awareness in patients assumed to be in a vegetative state, reducing the problem of misdiagnosis [27]. Functional neuroimaging, while not a replacement for behavioral assessment, could provide an additional objective measurement of regional cerebral activity offering further insight into the altered state of consciousness of these individuals.

Finally, future research in both healthy people and neurological patients should aim at shedding more light on two continuous processes which run in the background of consciousness-sustaining networks: (1) sensation and information about bodily states occasionally presents to consciousness mediated by the activity of the autonomic nervous system and neuro-hormonal pathways; (2) sensori-motor loops shape self/other awareness via continuous feedback from dynamic interaction with our external environment and social context.
